# Analysis of the Bacterial and Host Proteins along and across the Porcine Gastrointestinal Tract

**DOI:** 10.3390/proteomes7010004

**Published:** 2019-01-10

**Authors:** Johanna Tröscher-Mußotter, Bruno Tilocca, Volker Stefanski, Jana Seifert

**Affiliations:** Institute of Animal Science, University of Hohenheim, Emil-Wolff-Str. 6-10, 70567 Stuttgart, Germany; johanna.troescher@uni-hohenheim.de (J.T.-M.); brunotilocca@gmail.com (B.T.); Volker.Stefanski@uni-hohenheim.de (V.S.)

**Keywords:** metaproteomics, microbiome, pig

## Abstract

Pigs are among the most important farm animals worldwide and research to optimize their feed efficiency and improve their welfare is still in progress. The porcine intestinal microbiome is so far mainly known from sequencing-based studies. Digesta and mucosa samples from five different porcine gastrointestinal tract sections were analyzed by metaproteomics to obtain a deeper insight into the functions of bacterial groups with concomitant analyses of host proteins. Firmicutes (Prevotellaceae) dominated mucosa and digesta samples, followed by Bacteroidetes. Actinobacteria and Proteobacteria were much higher in abundance in mucosa compared to digesta samples. Functional profiling reveals the presence of core functions shared between digesta and mucosa samples. Protein abundances of energy production and conversion were higher in mucosa samples, whereas in digesta samples more proteins were involved in lipid transport and metabolism; short-chain fatty acids production were detected. Differences were also highlighted between sections, with the small intestine appearing more involved in carbohydrate transport and metabolism than the large intestine. Thus, this study produced the first functional analyses of the porcine GIT biology, discussing the findings in relation to expected bacterial and host functions.

## 1. Introduction

The mammalian gastrointestinal tract (GIT) is settled by a wide range of different microorganisms that coexist in a sensitive ecosystem and mutual cooperation, whereas bacteria predominate. Within the GIT, the microorganisms are not only living on the nutrients and energy out of the diet, but they also produce several substances such as vitamins, organic acids, secondary bile acids, and gases. Their products and the simple existence of microorganisms show an influence on host health, whereas changes in diet, environmental stress, and disease can change the microbiome within the GIT [1].

The pig (*Sus scrofa*) is one of the most important farm animals in today’s agroeconomy, with the swine industry expanding worldwide. Achieving balance between today’s meat production methods and animal welfare has proven to be difficult. To enhance the pig’s health situation—the production efficiency as well as the product quality—it is important to understand the intestinal environment, especially the interactions among microorganisms and between microorganisms and their host [2,3]. The predominant aim of intestinal microbiome investigations in farm animal sciences is to determine a balanced microbial composition to optimize animal health, performance, and pathogen resistance [4], but also to investigate which microbiome composition maximizes the benefits and minimizes the costs of animal husbandry [5]. Also, the feed industry is interested in establishing or preserving this microbiota by developing feed additives, diets, and other interventions [3,5]. In addition, the pig seems to be interesting for human medical purposes as it reveals similarities in size, immunobiology, distribution of lymphocytes [6], microbial ecosystems [7,8], physiology, and disease development to humans [9]. Thereby, pigs can potentially be used as model animals for human biology [10].

The important interface between the microorganism and the host in the GIT is the mucus which functions as a barrier between feed, microorganisms, and the animal [11]. It is produced by exocrine glands and the goblet cells, which are located in the mucosal epithelium and protects the former against injuries, but also against chemical and physical forces. Mucosa consist of a complex mixture of large glycoproteins (so-called mucins) water, electrolytes, separating epithelial cells, and enzymes, but also secreted immunoglobulins and antimicrobial molecules [12]. The mucus contains bacteria and cell debris, since the mucus is the first connecting site between host and bacteria. In previous years, the mucus-associated microbiota in pigs became of interest and were studied in regards to dietary effects and age-related changes [13,14,15,16,17,18,19]. The overall intestinal bacterial phyla in pigs are headed by Firmicutes, Bacteroidetes, Proteobacteria, and Spirochaetes, whereas Fibrobacteres, Actinobacteria, Tenericutes, Synergistetes, and Planctomycetes account for less than 1% of all 16S rRNA gene sequences [13,20,21]. 

Since more than a decade, metaproteomics has been used to examine microbial proteins in different sample types to identify and quantify metabolic proteins and the pathways they are involved in [22]. Microbial protein and host protein co-extraction is an intrinsic bias whose effect can only be minimized, not avoided [23,24]. Although it was tried to prioritize microbial protein extraction, co-extracted host proteins have been used to concurrently study the metabolic status of the host [6]. Thus, a benefit can be found in identifying the bacterial and host proteins in one run which gives new insights to both parts from exactly the same sample.

The present work attempted to detect the active bacterial fraction of the pig’s microbiome along five different sections (stomach, ileum, jejunum, cecum, and colon) by considering both luminal (digesta) and mucosal compartments of each section. A concomitant surplus is the identification and description of the porcine proteome to follow host functions.

## 2. Material and Methods

### 2.1. Animal Experiment and Sampling

All experiments and care of animals were approved by the local authorities (Regional Commission of Stuttgart, permit number: V308/13 TH) in accordance with the German Welfare Legislation. This study was generated in addition to the study of Heyer et al. [25], from which detailed trial operations can be taken. Pigs (German Landrace × Piétrain, initial body weight 54.7 kg ± 4.1 kg) were randomly assigned to four experimental diets. Diets were formulated to meet or exceed the animal’s nutrient requirement and differ among each other in the protein source and the calcium and phosphorous (CaP) levels. Two out of the four diets contain the low digestible (LD) corn-field peas meal as a protein source, whereas the remaining two diets comprise the high digestible (HD) corn-soybean meal as a protein source. Each of these dietary groups was further supplied with high and low CaP levels. Diets with high and low CaP level were formulated to contain respectively 120% and 66% of the requirement for 50–75 kg pigs [26].

After a feeding period of 9 weeks, including an adaptation of 19 days to the diets, one female pig per diet was anesthetized and euthanized by intravenous injection via the ear vein with pentobarbital (about 70 mg/kg BW, CP-Pharma, Handelsgesellschaft mbH, Burgdorf, Germany). Mucosae from the stomach (*Pars nonglandularis*), and both digesta and mucosae from jejunum (80 cm from the Plica ileocecalis), ileum (20 cm from the Plica ileocecalis), cecum, and the mid-colon were aseptically collected by scraping the mucosal layer from the tissue with a sterile glass slide and stored at −80 °C.

### 2.2. GC Analysis of Short Chain Fatty Acids

Concentration of short chain fatty acids (SCFA) in jejunal and cecal samples was analyzed by gas chromatography according to Wischer et al. (2013) [27]. Briefly, SCFA were directly measured in a gas chromatographer equipped with a flame ionization detector (HP 6890 Plus; Agilent, Waldbronn, Germany). GC-grade short chain fatty acids (Fluka, Taufkirchen, Germany) were used as internal standards. Measurements were performed in a capillary column (HP 19091F-112, 25 m × 0.32 mm × 0.5 μm) by following the given program: 80 °C, 1 min; 155 °C in 20 °C/min; 230 °C in 50 °C/min., constant for 3.5 min., carrier gas: helium. Short-chain fatty acid concentration is scored as referred to kilogram sample.

### 2.3. Sample Preparation

The sample preparation was carried out after the method of Apajalahti et al. [28]. Each sample containing 5 g of fresh substance was resuspended in 10 mL of washing buffer. The following steps of protein extraction, quantification, digestion, and peptide purification were performed as previously described by Tilocca et al. [29].

### 2.4. LC-MS/MS Analysis

Purified peptides were analyzed using an EASY-nLC 1000 system coupled to a Q-Exactive Plus mass spectrometer (Thermo Fisher Scientific, Waltham, MA, USA). Peptides were injected and separated into an EASY-Spray analytical column (2 µm, 100 Å PepMap RSLC C18, 25 cm × 75 µm, Thermo Fisher Scientific) using the following 235 min gradient: 2–10% solvent B in 100 min; 10–22% solvent B in 80 min; 22–45% solvent B in 55 min; 45–90% solvent B in 5 min, 15 min isocratic at 90% solvent B; 90–2% solvent B in 1 min; re-equilibration at 2% solvent B for 40 min. Solvent A contained 0.5% acetic acid and solvent B consisted of 0.5% acetic acid in ACN/H_2_O (80/20). The flow rate was 250 nL/min and the column temperature was constantly kept at 35 °C.

The MS spectra (*m/z* = 300–1600) were ascertained at a resolution of 70,000 (*m/z* = 200) using a maximum injection time (MIT) of 50 ms and an automatic gain control (AGC) value of 1 × 10^6^. The internal calibration of the Orbitrap analyzer was conducted consulting lock-mass ions from ambient air following the method of Olsen et al. [30]. The 10 highest peptide precursors were used for data dependent MS/MS spectra. Therefore, high energy collision dissociation (HCD) fragmentation was used with the following settings: resolution 17,500; normalized collision energy of 25; intensity threshold of 2 × 10^5^. For fragmentation, only ions with charge states between +2 and +5 were chosen. Therefore, an isolation width of 1.6 Da was set. AGC was adjusted at 1 × 10^6^ whereas MIT was set at 50 ms for each MS/MS scan. To prevent further fragmentation, it was decided to eliminate fragmented precursor ions for 30 s within a 5 ppm mass window.

### 2.5. Data Analysis

The raw files from the mass spectrometric measurements were analysed by MaxQuant (v 1.5.1.2, Max Planck Institute of Biochemistry, Munich, Germany) using the database consisting of sequences of the *Sus scrofa* genome (61,019 entries, March 2016) and an in-house database of bacterial proteins (14,535 entries, October 2015) identified by a two-step search approach in a previous study analyzing 84 porcine fecal samples [31]. Protein grouping node was activated with the default software settings. The data analyses failed to include protein entries from dietary intake, leading to non-identified proteins, which are probably dominant in the digesta samples.

Phylogenetic distribution of the bacterial proteins was assessed on the basis of the identified peptides with Unipept [32]. This tool provides the phylogenetic assessment of the bacterial community up to strain level depending on amino acid sequence homologies. In the present study, protein identification is done at phylum and family level as deeper taxonomic levels are omitted due to a low peptide-to-protein ratio and the risk of false positive identification. Calculation of alpha diversity and statistical evaluation was done with Primer-E (v. 6, primer-e, Auckland, New Zealand), by first standardizing the peptide datasets and afterwards creating a lower triangular resemblance matrix (resemblance measure S17 Bray Curtis similarity). The functional classification of the bacterial proteins was performed by the categorization into COG classes through the WebMGA online tool. [33]. Proteins descending from the pig were categorized and illustrated using proteomaps [34].

### 2.6. PRIDE Accession

The mass spectrometry proteomics data have been deposited to the ProteomeXchange Consortium via the PRIDE [35] partner repository with the dataset identifier PXD011360 (login Username: reviewer73047@ebi.ac.uk, Password: a1lkk6Ch)

## 3. Results and Discussion

### 3.1. General Protein Results

A total of 2951 different bacterial proteins were identified, with a higher number of bacterial proteins recovered in the digesta (2917) than in the mucosa (973). The overall distribution of the bacterial proteins is shown in Figure S1. The co-identification of porcine proteins revealed 4550 hits in total (Table S1).

**Figure 1:**
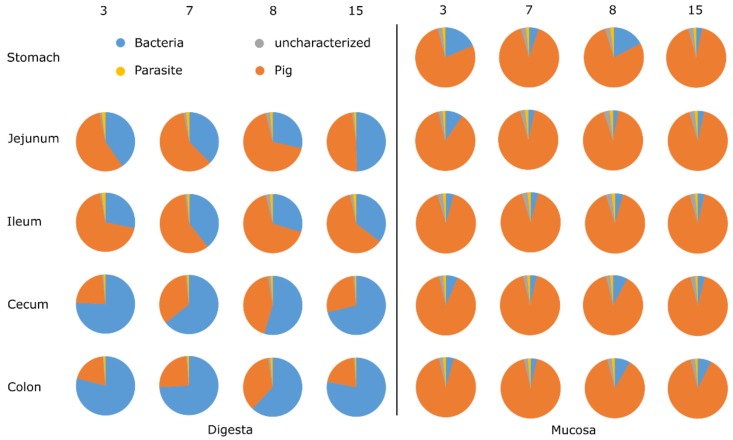
Relative distribution of proteins in digesta and mucosa samples of the pig into parasite, bacteria, pig, and uncharacterized proteins across gastrointestinal tract sections and compartments in four animals. Numbers 3, 7, 8, and 15 are pig identifiers.

An initial interest was the investigation of the protein distribution of all identified proteins in gut sections and compartments. The proteins of digesta samples from jejunum and ileum were by the majority originating from the pig. Bacterial proteins were underrepresented here as in these sections the number and diversity of bacterial cells is lower compared to cecum and colon. Thus, the sole limitation of available biomass can be the reason for a limited identification of proteins with mass spectrometry. In the distal gut sections, proteins mainly originated from bacteria and only sparsely from the host (Figure 1, Table S1). Parasite and uncharacterised proteins were low in abundance in all samples.

Pig proteins were the majority in all mucosa samples (>75% pig protein), with highest protein counts in the colon mucosa (Figure 1). Even though the mucosal compartments revealed higher total numbers of proteins, all mucosa samples showed much lower counts of bacterial proteins than the digesta samples (973 vs. 2917 proteins). The highest counts of bacterial proteins were found in cecum digesta samples with an average of 1384 proteins.

**Figure 2:**
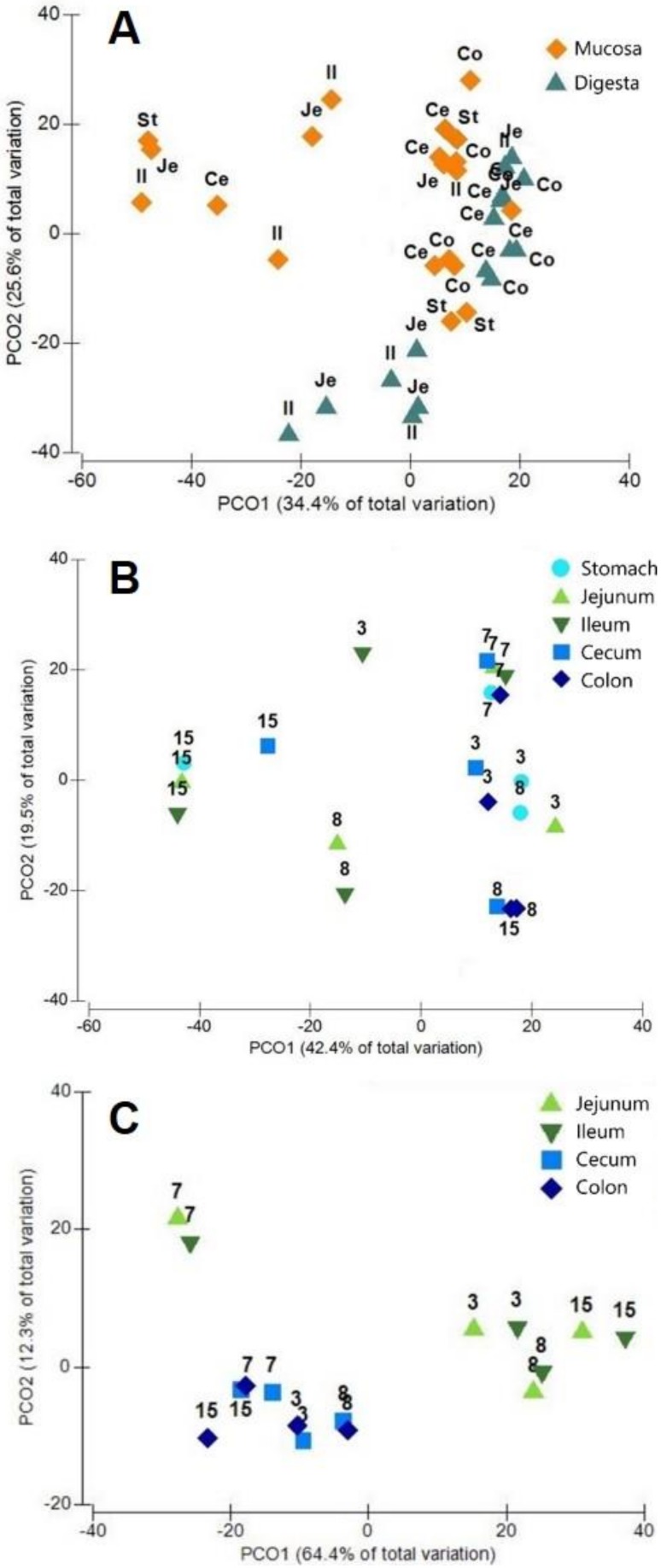
Sample ordination to discriminate the protein distribution between mucosa and digesta samples (**A**), within the mucosa (**B**) and the digesta (**C**) samples. Principal Coordinate Analysis (PCoA) plots were drawn from protein data using S17 Bray Curtis similarity. The percentage represents the contribution of the principal component to the difference in sample composition. Points of different colors and shapes represent samples of different groups, and the closer the two sample points are, the more similar the composition of the samples species is.

The overall comparison between the protein results of digesta and mucosa showed a separation of the datasets (Figure 2A). In addition, mucosal samples were grouped according the animal and not based on the section. Digesta samples showed a separation based on both factors, animal and section. The separation between small intestine (jejunum and ileum) and large intestine (cecum and colon) (Figure 2B,C) was approved with a significant value of *p* = 0.0004 for the comparison between ileum and colon samples.

### 3.2. Phylotypes of Bacterial Proteins in Digesta

The alpha diversity of the digesta samples showed the highest diversity in cecum and colon samples with Shannon indexes of 2.7 vs. 2.4 in small intestine samples (Figure S2A). The lower diversity in the ileum is also described in other microbiota studies where amplicon sequencing was used [17,21]. This difference is caused by the luminal environment in the small intestine, where pH conditions, higher oxygen levels, and a fast digesta passage [36] may be a challenging site for the fermenting bacteria. The longer retention time in the large intestine favors the fibre fermentation process, which leads to an enhanced energy gain for bacteria using these substrates and the corresponding fermentation products, respectively [37].

**Figure 3:**
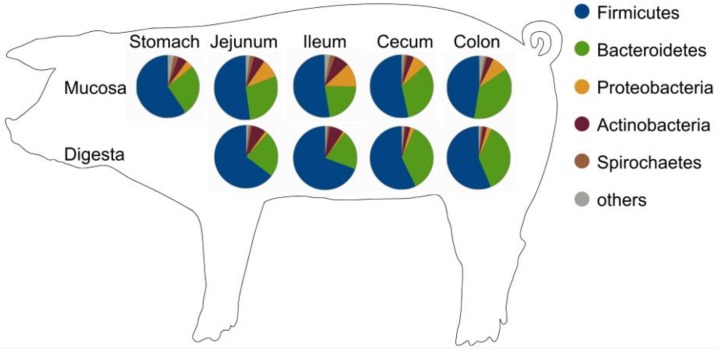
(**A**) Averaged distribution of bacterial phyla in mucosa and digesta of the swine intestine. (**B**) Bacterial families (>1%) found in digesta and (**C**) mucosa of all animals in relative abundance (%) based on the number of peptides. Numbers of referring peptides are given beneath each bar. The fifth bar represents an average (Ø) of the section amongst all animals.

The major phyla identified based on the bacterial proteins in digesta along the GIT were Firmicutes followed by Bacteroidetes and Actinobacteria (Figure 3 and Figure S3). The predominant abundance of families belonging to Firmicutes and Bacteroidetes is equivalent to studies based on amplicon sequencing [16,17,20,21]. Dominating families affiliated to the proteins were Prevotellaceae, Clostridiaceae and Lactobacillaceae (Table 1 and Table S2).

Proteins belonging to Firmicutes showed higher peptide counts in the small intestine (jejunum 64.7%; ileum 69%) compared to the cecum and the colon. This trend was also shown based on operational taxonomic unit (OTU) counts in young pigs [17], whereas the opposite was described in 28-day-old piglets [16]. Here, proteins of Lactobacillaceae, Clostridiaceae, and Veillonellaceae dominate this phylum in all sections, which is in line with other recent pig microbiota studies [15,17,21] (Figure 3B). Lactobacillaceae proteins were considerably high in the small intestine (jejunum 15%; ileum 18%) compared to the large intestine (cecum 9%, colon 6%). Proteins of the family Clostridiaceae showed higher counts of assigned peptides in the ileum (16%). Peptides of Veillonellaceae decreased continuously by half from jejunum (11%) to the colon (5%).

Proteins of Bacteroidetes were highly abundant, whereas its average occurrence was much higher in the large intestine (36%) compared to the small intestine (22%). The majority of proteins belonged to Prevotellaceae, which were increasing from the small (20%) to large intestine (27%). Again, theses changes in abundance along the GIT fits with other findings [17].

In the present study, Actinobacteria proteins defined mainly by the ones from Bifidobacteriaceae were found in all sections of all animals, with greater average abundances in the small intestine (6%) compared to an abundance below 1% in the colon. Thus, Actinobacteria appear to be the third most abundant phylum in this metaproteomic analyses. This is in contrast to previous sequencing studies where Proteobacteria is at the third position [17,21]. In an accompanied study, copy numbers of *Bifidobacterium* sp. were found mainly in the jejunum and caecum, but not in the colon, which fits in with the proteomic results [38]. Bifidobacteriaceae are producing acids from a wide range of carbohydrates via the specific metabolic pathway called the “bifid shunt” [39]. Feeding dietary fibers leads to a lowered pH of the gut content and causes an environment, which is favorable for *Bifidobacterium* sp. In several studies, high fibre diets cause an increase of beneficial bacteria such as Bifidobacteriaceae in the cecum [40] and support the intestinal epithelial morphology [15]. Both contribute to the pigs’ GIT health and lead to a higher defensive power against harmful organisms [40].

### 3.3. Phylotypes of Bacterial Proteins in Mucosa

The alpha diversity of the mucosal samples was characterized by Shannon indexes of around 2.4 along the small intestine and the cecum, and an average index of 2.6 in colon samples (Figure S2B). No clear increase in diversity was found compared to lumen samples, although this was estimated on the basis of other microbiota studies. There, Shannon indexes between the lumen and mucosa samples of the small intestine almost doubled [17,20]. One reason for this discrepancy is probably caused by the lower number of identified bacterial proteins in the mucosal samples (Figure 1).

The majority of the proteins from mucosal samples belonged to Firmicutes with 59% in the stomach and 53% to 47% in subsequent sections (Figure 3, Table 2 and Table S3). Peptides of Lactobacillaceae had the highest counts within all peptides belonging to this phylum on the mucosal site. A longitudinal change was observed with higher abundances in the stomach and small intestine (about 19%) and a reduction by more than half in the colon (8%). Peptides of Clostridiaceae showed a reverse abundance pattern ranging from 4% in the stomach to 11% in the colon. In contrast, peptides of Lachnospiraceae were equally distributed along the mucosal samples of young pigs.

The abundance of peptides assigned to Bacteroidetes changed from stomach (27%) to the small intestine (jejunum, 29%; ileum, 22%) and increased in the large intestine (37%) (Table 2). Peptides of Prevotellaceae showed the highest abundance belonging to Bacteroidetes in the mucosal samples. A decrease of Prevotellaceae peptides was seen from stomach (20%) to ileum (15%), with the highest abundances found in the large intestine (27%). The dominance of the *Lactobacillus* and *Prevotella* was also described by Mann et al. [14] which characterized the mucus-associated microbiota along the GIT. Interestingly, ileum samples of mucosa and digesta decreased in Prevotellaceae peptide numbers and simultaneously increased in Lactobacillaceae peptides. This again may derive from the tolerance of several genera of the latter to bile [41], whereas *Prevotella* cells may be more sensitive to this secretion.

The highest peptide hits in the ileum were assigned to Proteobacteria (12%), whereas the lowest were found in the stomach (4%) (Figure 3). Pasteurellaceae was the dominating family of this phylum, with higher peptide hits in the ileum. Pasteurellaceae are a large family including pathogenic and commensal organisms [42]. They are oxidase producing aerobic to facultative anaerobic bacteria living on mucus layers of the respiratory-, genital- and gastrointestinal tract [42]. Higher abundances in the mucus of the large intestine enhance the assumption that in here, Pasteurellaceae can alter to anaerobic respiration as oxygen levels are decreasing from proximal to distal sections [42]. In contrast, Proteobacteria were identified to contribute up to 50% of cecal mucosa transcripts with Helicobacteraceae as predominant family in growing pigs [19]. In piglets, this bacterial family has been observed to dominate the mucosa of the small intestine [16].

### 3.4. Functional Annotation and Distribution of Bacterial Proteins in Digesta and Mucosa

Classification of the identified proteins into COG classes reveals that major functions of the bacterial community of both digesta and mucosa were linked to energy production and conversion, translation and carbohydrate transport and metabolism. The overall distribution of the functions is relatively robust between the sections and animals in the digesta samples, whereas mucosal samples showed a host-dependent effect across all GIT sections (Figures S5 and S6).

Proteins related to energy production and conversion were very similar between all animals along all sections with a higher relative abundance in the mucosa (23–31%) than in the digesta (23–25%), matching the results of another study [24]. In mucosa samples, these proteins were mainly assigned to the oxidative phosphorylation, followed by carbon metabolism and carbon fixation. Proteins from digesta samples were additionally sorted into pyruvate metabolism. Main proteins belonging here were rubrerythrin and succinate dehydrogenase/fumarate reductase followed by components of the pyruvate ferredoxin oxidoreductase (Tables S4 and S5). Rubrerythrin was assigned to different bacterial families within the Clostridiales. For the reduction of fumarate especially *Prevotella* was assigned. These phylogenetic assignments are in accordance with a rat microbiome analyses [24].

Proteins sorted into the translation cluster on average remained quite alike along all sections. GTPases-translation elongation factors and a wide range of ribosomal proteins (L2, S3) dominated this cluster (Tables S4 and S5).

More proteins of the carbohydrate transport and metabolism group were counted in the small intestine than in the large intestine (Figure S5) and mainly belong to glycolysis and gluconeogenesis. Main proteins within this group were glyceraldehyde-3-phosphate dehydrogenase/erythrose-4-phosphate dehydrogenase (amongst others, produced by *Anaerotruncus*, *Pseudomonas* and *Paraprevotella*) and ABC-type sugar transport systems (ATPase components).

Proteins related to the biosynthesis of amino acids were also highly counted with an average contribution of 5% in digesta samples and a section-dependent abundance of 2% in colon mucosa and 9% in cecal mucosa samples. The relative abundance of proteins assigned to lipid transport and metabolism was on average higher in digesta samples (3%) than in mucosa samples (2%). A dominating protein assigned to this cluster was acyl-CoA dehydrogenases, followed by acetyl-CoA acetyltransferase.

**Figure 4:**
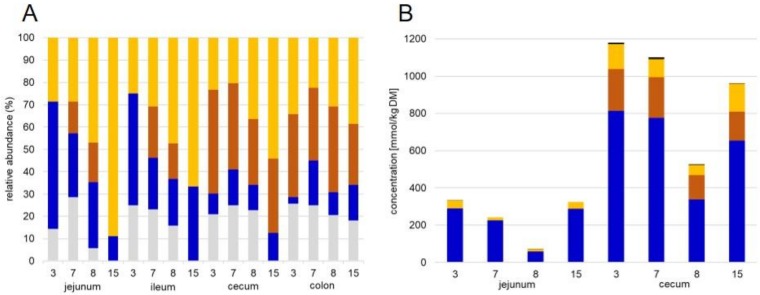
Distribution of proteins involved in single short chain fatty acid (SCFA) biosynthesis (**A**) and measured concentrations of single SCFA (**B**) in digesta samples shown for each section and pig. Color code is equal for A and B: light grey: formate, blue: acetate, dark orange: propionate, light orange: butyrate, grey: valerate. Proteins were sorted according to Polansky et al. [43] (Table S4).

Proteins involved in the formation of SCFA were mainly identified in digesta samples (Figure 4A) with variable distributions of proteins belonging to formate, acetate, propionate, and butyrate formation. Protein sorting was done as described by Polansky et al. [43] (Table S6). The taxonomic affiliation of these proteins showed a dominant contribution of Clostridiales and Veillonellales to produce butyrate in the small and large intestine, which is complemented in the distal gut section by members of Spirochaetes. Proteins involved in propionate formation are expressed mainly by Acidaminococcales, Veillonellales and Selenomonadales and to a less extent by Bacteroidales. In the small intestine, acetate forming proteins were affiliated with Bifidobacteriales and Veillonellales, whereas in the large intestine Bacteroidales and Clostridiales proteins were found. These phylogenetic findings fit the meta-analysis of the core swine gut microbiota where the functional role of the phylotypes were just discussed based on the presence of the 16S rDNA sequencing data [21]. Formate biosynthesis seemed to be done by Coriobacteriales and Clostridiales in the small intestine and by Clostridiales and Fusobacteriales in the large intestine (Table S6). The total concentration of SCFA in the digesta samples showed almost 4 times higher values from jejunum to cecum (Figure 4B). Similar ratios were also measured in other studies and discussed with the increased bacterial diversity and fermentation capacity in the cecum compared to small intestine [44]. Among all SCFA drastic changes were found for propionate and butyrate which increased from 0.9 to 182 mmol/kg DM and 26 to 108 mmol/kg DM, respectively (Figure 4B). This is in accordance with the change in diversity of bacterial phylotypes involved in these fermentation activities and was described by others as well [44].

### 3.5. Animal Proteins

**Figure 5:**
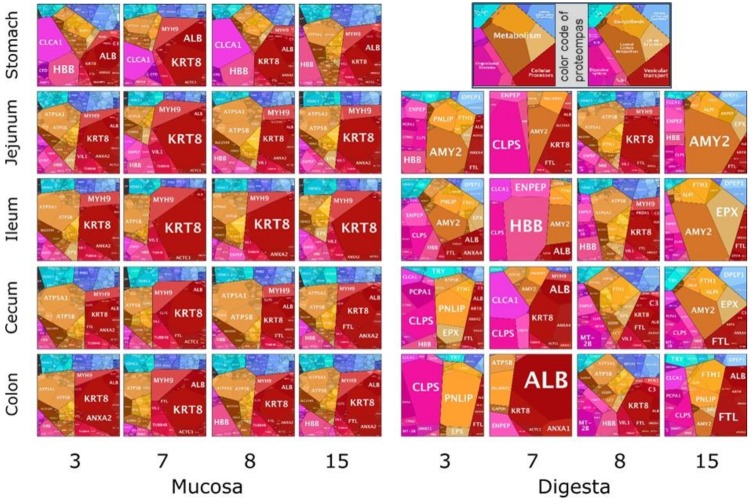
Proteomaps of animal mucosa (**left**) and digesta (**right**) proteins per animal and section. Numbers 3, 7, 8, and 15 are pig identifiers. Abbreviations of protein groups are explained in Section 3.5.

The mass spectrometric measurements of all samples allowed an additional identification of pig proteins using a database of the *Sus scrofa* genome. The general distribution of pig proteins was more homogeneous along the sections in the mucosa samples compared to the digesta samples (Figure 5).

Mucosal proteins were mostly assigned to metabolism and organismal systems of the host cells. Cellular processes was the one dominating general cluster of all sections’ mucosa. Within this cluster, proteins related to exosomes where the membrane associated ones such as keratin 8 (KRT8), annexin A2 (ANXA2), and albumin (ALB) predominate this cluster, followed by tight junction proteins (myosin, heavy chain 9 (MYH9)). In lower abundance, cytoskeletal proteins (tubulin beta 4B (TUBB4B)) were found in all sections. They are all important for the stabilization of the cytoskeleton and the cell shape. In general, proteins belonging to the cluster of organismal systems were more represented in the stomach sample and continuously less in protein counts within the distal sections. Proteins binned to haemoglobin beta (HBB) were quite high in the stomach samples. However, these polygons decreased towards the ilea and increased again in the colon sections. In the general cluster of genetic information processing, functional proteins related to the mitochondrial biogenesis (prohibitin 2 (PHB2)) and translation factors (mitochondrial translation elongation factor (TUFM) and eukaryotic translation elongation factor (EEF2, EEF1A1)) were found. This cluster was of smaller size in all ileum samples. The general cluster of metabolism proteins was dominated by proteins that were mainly assigned to oxidative phosphorylation such as subunits of ATP synthase and were followed by transport related proteins (e.g., solute carrier family 25 (SLC25A5)). The capacity to take up microbial products such as lactate and other SCFA were seen by the identification of monocarboxylic acid transporters (MCT1, MCT4), where two to three times more peptides were counted in the samples of the large intestine than in the small intestine. This trend of abundance matches to expression data from the human GIT [45]. The general cluster of environmental information processing was dominated by a subcluster related to the calcium signalling pathway (voltage dependent anion channel 1 (VDAC1)), which increased from stomach to cecum.

Proteomaps of the digesta samples showed a more heterogeneous picture than the mucosa samples since functions of proteins differed widely between sections and animals. Main general clusters were those related to organismal systems, metabolism and for some animals also cellular processes were predominant here (Figure 5). Main proteins of the organismal information processing cluster in digesta samples were related to hemoglobin, pancreatic secretion (PCPA1), reninangiotensin system (RAS, ENPEP), fat digestion, and absorption functions (colipase, CLPS). For animals 3, 8, and 15, this cluster increased from jejunum to colon, except for animal 7. Cellular process proteins were dominantly assigned to exosomes (KRT8, ANXA2, ALB) such as in mucosa samples. The general cluster of metabolism functions also dominated several sections of animal proteomaps. The lipid and steroid metabolism was one dominating sub cluster, showing a greater number of assigned proteins in the large intestine. These trends of protein abundances were unexpected as the pancreatic lipase (PNLIP) hydrolyses dietary fats into fatty acids and enters the duodenum via pancreatic secretion [46]. Thus, a higher abundance is expected in the small intestine. Also higher present were proteins annotated to carbohydrate metabolism such as pancreatic amylase (AMY2), which were elevated in the jejunum and ileum section.

The heterogeneous results obtained with the digesta samples indicate a highly variable matrix which challenges the interpretation of the animals’ metabolism based on the proteome. In this view, we also need to consider the differences in the dietary treatments of each animal, since it is most likely affecting the host response, thus the resulting proteome. Unfortunately, the lack of replicates per dietary treatment doesn’t allow us to deduce any satisfactory conclusions out of this. Animal proteins from the mucosa are more likely an adequate site for the investigation of host functions within a specific section. Here, host cells are involved in the respective functions such as the secretion of digestive enzymes or the uptake of feed compounds. The response and the potential changes of the proteomes are due to the different conditions in the microenvironment.

## 4. Conclusions

The current study employs a metaproteomic approach to investigate the porcine microbial community associated with diverse GIT sections, both in its mucosal and luminal compartment. The obtained results highlight a clear alteration between the small and the large intestine, which was evident at the bacterial phylogenetic level and by the distribution of functional protein clusters. This underlines the physiological differences between these two segments. Bacterial proteins in mucosa and digesta differed between sections of the pig in phylogeny and protein functions. A higher diversity of bacterial proteins was found in digesta samples compared to mucosa samples, albeit this might be an effect of a lower protein identification rate on the mucosal site. In general, from proximal to distal sections, more proteins and peptides were found. The metaproteomics approach is assumed to be the “keystone to ecosystematic studies” of environments and their associated microbial communities. Besides the sole consideration of the prokaryotic proteins in an organismal sample, this study showed the benefit to use the so far interference proteins of the host to reveal insight into the host metabolism. However, this method shows multiple challenges within several steps of the analytical workflow. Until now, a lot of information is lost by the data analysis process, since not all proteins or peptides can be identified and annotated to a function or phylogeny. Nevertheless, with this possible output, the present study gives us the first glimpse into the active microbiome of the porcine GIT and the host proteins. This may help us to identify key players or biomarkers as targets to design therapeutic intervention systems for various fields of application. Nevertheless, we retain that further investigations involving a higher number of animals and an integrative approach with other *Omics* methods (e.g., metagenomics or metatranscriptomics) will further facilitate the understanding and interpretation of the biology of the pigs’ gut and its associated microbial communities.

## Figures and Tables

**Table 1:** Relative abundances of bacterial families identified based on peptide sequences in digesta samples along the gastrointestinal tract sections as average values for all four animals (Ø) and standard deviation (SD).

	Jejunum		Ileum		Caecum		Colon	
	Ø	SD	Ø	SD	Ø	SD	Ø	SD
**Actinobacteria**								
Atopobiaceae	0.3	0.5	0.0	0.0	0.0	0.0	0.0	0.0
Bifidobacteriaceae	5.7	2.9	5.6	3.5	1.1	0.7	0.3	0.6
Streptomycetaceae	0.3	0.5	0.0	0.0	0.0	0.0	0.0	0.0
**Bacteroidetes**								
Bacteroidaceae	1.2	1.5	1.0	1.7	5.9	2.0	5.0	1.2
Porphyromonadaceae	0.6	0.6	0.4	0.6	2.5	0.5	2.6	0.4
Prevotellaceae	20.7	19.9	17.7	18.3	26.0	3.9	27.2	3.1
Rikenellaceae	0.0	0.0	0.0	0.0	0.3	0.6	0.7	0.7
**Firmicutes**								
Acidaminococcaceae	0.9	0.5	1.6	1.3	2.1	0.3	2.4	0.3
Clostridiaceae	13.0	6.0	15.7	6.4	10.4	2.2	11.6	3.3
Erysipelotrichaceae	1.7	1.0	1.9	1.2	1.1	0.7	1.2	0.7
Eubacteriaceae	1.3	0.8	1.7	1.1	2.5	0.7	2.3	0.8
Lachnospiraceae	4.7	0.8	3.8	1.2	7.8	1.2	8.7	0.9
Lactobacillaceae	15.0	7.8	17.7	10.7	8.9	2.0	6.4	1.4
Oscillospiraceae	0.5	0.9	0.4	0.6	1.0	1.1	1.1	1.1
Paenibacillaceae	0.3	0.6	0.0	0.0	0.0	0.0	0.0	0.0
Peptostreptococcaceae	1.5	1.5	1.8	1.9	0.7	0.7	0.9	0.9
Ruminococcaceae	3.0	1.1	2.7	1.8	7.6	2.5	6.9	3.3
Selenomonadaceae	8.9	3.3	8.9	3.2	5.8	2.1	5.6	2.0
Streptococcaceae	1.7	1.1	2.1	1.7	1.7	1.6	2.0	1.7
Veillonellaceae	10.5	6.3	8.8	5.7	5.6	2.5	4.8	2.0
**Proteobacteria**								
Pasteurellaceae	0.3	0.5	0.0	0.0	0.0	0.0	0.0	0.0
**Spirochaetes**								
Brachyspiraceae	0.8	0.8	0.6	1.0	0.0	0.0	0.0	0.0
Leptospiraceae	0.3	0.5	0.0	0.0	0.0	0.0	0.3	0.4
**other**	6.9	2.1	7.8	1.0	9.0	1.7	10.1	0.3
**no. of peptides**	192	46.2	176	60.1	509	75	486	95

**Table 2:** Relative abundances of bacterial families identified based on peptide sequences in mucosal samples along the gastrointestinal tract sections as average values for all four animals (Ø) and standard deviation (SD).

	Stomach		Jejunum		Ileum		Caecum		Colon	
	Ø	SD	Ø	SD	Ø	SD	Ø	SD	Ø	SD
**Actinobacteria**										
Atopobiaceae	0.4	0.7	0.5	0.9	0.5	0.9	0.8	0.8	0.4	0.7
Bifidobacteriaceae	1.2	1.3	0.0	0.0	1.6	2.7	0.0	0.0	0.4	0.7
Coriobacteriaceae	0.0	0.0	0.0	0.0	1.6	2.7	0.0	0.0	0.0	0.0
Eggerthellaceae	0.0	0.0	0.0	0.0	0.0	0.0	0.3	0.6	0.7	0.7
Streptomycetaceae	1.9	2.6	4.6	3.7	4.1	1.5	2.3	1.1	1.9	0.6
**Bacteroidetes**										
Bacteroidaceae	2.4	2.2	3.5	4.1	3.8	3.8	5.4	4.4	5.0	2.2
Flavobacteriaceae	0.0	0.0	0.0	0.0	0.0	0.0	1.7	1.8	1.5	1.5
Porphyromonadaceae	0.6	0.6	0.7	1.3	0.0	0.0	1.5	1.7	2.2	1.3
Prevotellaceae	20.3	7.8	19.8	4.5	14.7	4.3	22.3	8.5	26.5	6.3
Rhodothermaceae	1.6	2.7	4.1	4.1	3.6	2.3	1.9	1.5	2.2	0.6
Rikenellaceae	0.8	1.3	0.0	0.0	0.0	0.0	0.5	0.8	0.0	0.0
**Firmicutes**										
Acidaminococcaceae	0.7	0.7	1.2	1.3	0.5	0.9	0.8	0.8	1.1	0.7
Anaerolineaceae	0.0	0.0	0.0	0.0	0.0	0.0	0.3	0.6	0.7	0.7
Clostridiaceae	4.3	3.6	5.7	4.9	6.8	10.6	5.8	4.5	10.5	7.1
Erysipelotrichaceae	0.8	0.8	0.5	0.9	0.5	0.9	0.7	1.2	3.3	1.5
Eubacteriaceae	1.1	1.1	1.2	2.1	0.0	0.0	1.5	1.7	3.2	2.1
Lachnospiraceae	7.7	3.4	9.8	1.8	8.4	3.8	8.9	3.1	7.5	2.0
Lactobacillaceae	19.0	14.4	18.0	18.7	19.9	15.0	17.9	8.9	8.0	3.1
Oscillospiraceae	0.3	0.5	0.0	0.0	1.1	2.0	1.3	1.5	0.0	0.0
Paenibacillaceae	0.0	0.0	1.0	1.0	0.9	1.5	0.3	0.6	0.7	0.7
Peptoniphilaceae	0.0	0.0	0.0	0.0	0.0	0.0	0.3	0.6	0.7	0.7
Peptostreptococcaceae	0.0	0.0	0.0	0.0	0.0	0.0	1.9	1.5	0.7	0.7
Ruminococcaceae	2.5	2.3	3.0	3.3	4.3	4.3	3.7	3.7	3.8	1.7
Selenomonadaceae	9.3	7.9	2.0	2.4	1.5	2.6	1.6	1.7	2.3	1.8
Streptococcaceae	2.3	2.4	4.6	3.7	4.1	1.5	2.7	0.9	1.5	1.0
Symbiobacteriaceae	0.0	0.0	0.0	0.0	0.0	0.0	0.3	0.6	0.7	0.7
Veillonellaceae	10.2	6.9	3.7	4.1	4.8	4.8	5.4	3.8	2.4	3.5
**Nitrospirae**										
Nitrospiraceae	0.4	0.7	0.5	0.9	0.5	0.9	0.5	0.8	0.4	0.7
**Proteobacteria**										
Alcaligenaceae	0.0	0.0	2.3	3.9	0.0	0.0	0.0	0.0	0.0	0.0
Anaplasmataceae	0.0	0.0	2.8	3.7	0.5	0.9	0.5	0.8	0.8	0.8
Burkholderiaceae	0.0	0.0	0.0	0.0	0.0	0.0	0.0	0.0	0.4	0.7
Comamonadaceae	0.4	0.7	0.0	0.0	0.5	0.9	0.5	0.8	0.4	0.7
Enterobacteriaceae	0.0	0.0	0.0	0.0	1.1	2.0	1.9	1.5	0.7	0.7
Helicobacteraceae	0.8	1.3	2.3	3.9	0.9	1.5	0.5	0.8	1.1	1.2
Pasteurellaceae	1.4	1.4	1.5	1.7	8.0	6.1	3.3	2.0	3.4	0.5
Pelagibacteraceae	0.0	0.0	0.0	0.0	0.0	0.0	0.3	0.6	0.7	0.7
Vibrionaceae	0.0	0.0	0.0	0.0	1.1	2.0	0.0	0.0	0.4	0.7
**Spirochaetes**										
Brachyspiraceae	0.0	0.0	0.0	0.0	1.1	2.0	0.0	0.0	0.0	0.0
Leptospiraceae	2.4	2.3	2.3	2.9	1.6	1.9	1.5	1.7	1.1	1.2
**Synergistetes**										
Synergistaceae	0.4	0.7	0.5	0.9	0.5	0.9	0.5	0.8	0.4	0.7
**Tenericutes**										
Mycoplasmataceae	1.9	2.6	0.5	0.9	1.6	2.7	0.3	0.6	1.9	0.6
**other**	5.0	5.0	3.5	6.0	0.0	0.0	0.0	0.0	0.0	0.0
**no. of peptides**	100.8	62.3	44.0	36.3	29.0	12.8	50.8	17.8	58.0	14.1

## Supplementary Materials

The following are available online at http://www.mdpi.com/2227-7382/7/1/4/s1. Table S1: Number of proteins identified with Sus scrofa and bacteria database. Including taxonomic information revealed by Unipept searches. Table S2: Relative abundances of bacterial families in digesta samples based on peptide information along the GIT sections in each animal and on average Ø, standard deviation (SD). Table S3: Relative abundances of bacterial families in mucosa samples based on peptide information along the GIT sections in each animal and on average Ø, standard deviation (SD). Table S4: Bacterial proteins identified in all digesta samples and sorted according to COG classification Table S5: Bacterial proteins identified in all mucosa samples and sorted according to COG classification. Table S6: Phylogenetic affiliation of proteins belonging to SCFA biosynthesis in digesta and mucosa samples along the GIT section.

## Abbreviations

GIT: gastrointestinal tract; SCFA: short chain fatty acids
