# Supplementary material for: Analysis of the Bacterial and Host Proteins along and across the Porcine Gastrointestinal Tract
Source: Proteomes. 2019 Jan 10;7(1):4. doi: 10.3390/proteomes7010004 (PMC6473940; doi:10.3390/proteomes7010004)
Supplement: Supplementary file 1 [file proteomes-07-00004-s001.zip › proteomes-385353-supplementary-final version/proteomes-385353-supplementary-final version.docx]

# Supplementary materials

**Analysis of the bacterial and host proteins along and across the porcine gastrointestinal tract**

Tröscher-Mußotter, J.B. ^1^, Tilocca, B. ^1^, Stefanski, V. ^1^, Seifert, J. ^1,^*

^1^ Institute of Animal Science, University of Hohenheim, Emil-Wolff-Str. 6-10, 70567 Stuttgart, Germany


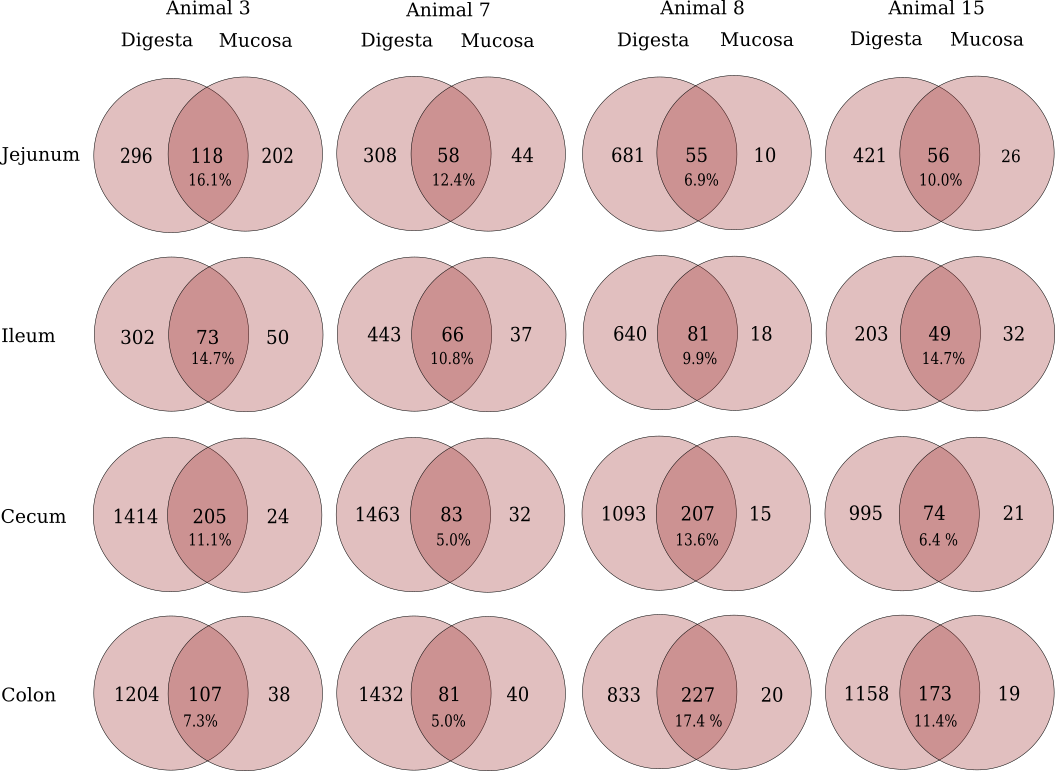


Figure S1: Distribution of bacterial proteins identified from mucosal (right circle) and digesta samples (left circle) as well as shared proteins (middle).


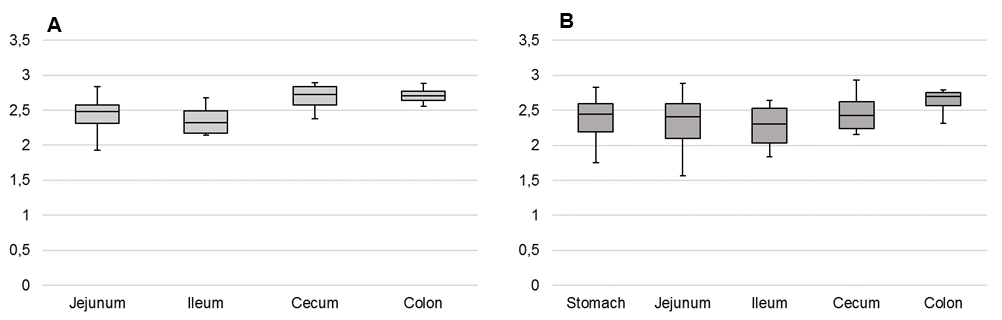


Figure S2: Shannon diversity index calculated for digesta (A) and mucosa (B) samples.


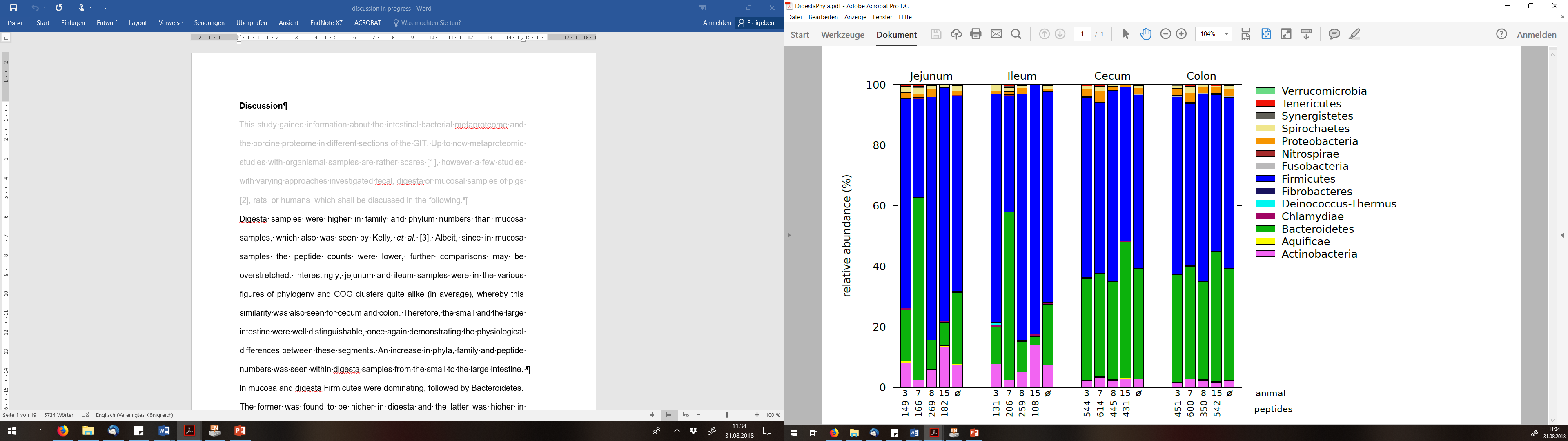


Fig S3 Distribution of bacterial phyla in digesta samples.


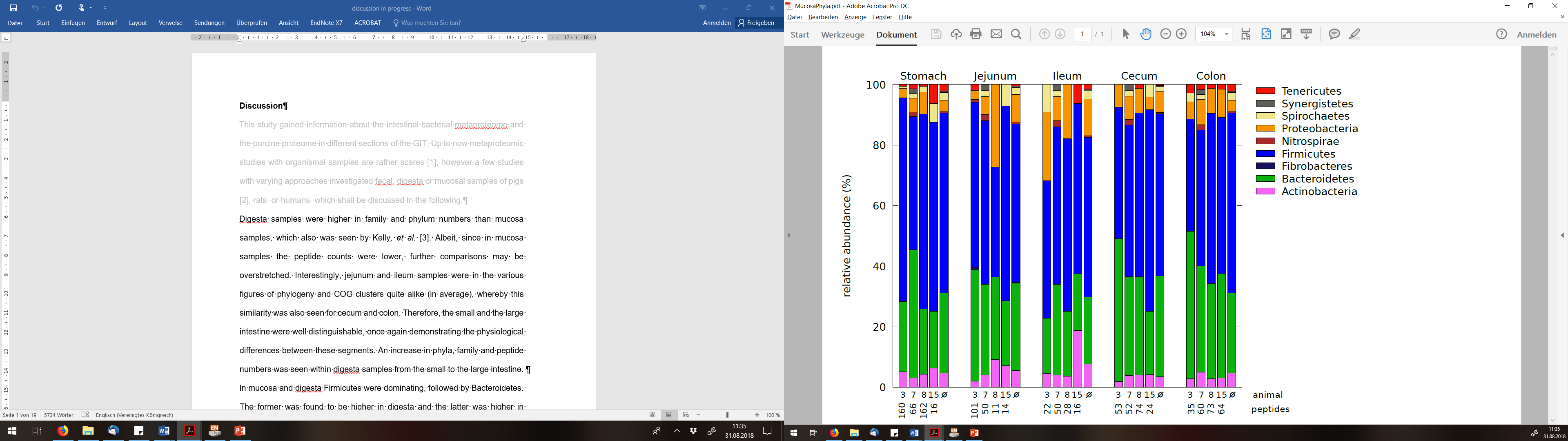


Fig S4 Distribution of bacterial phyla in mucosa samples.


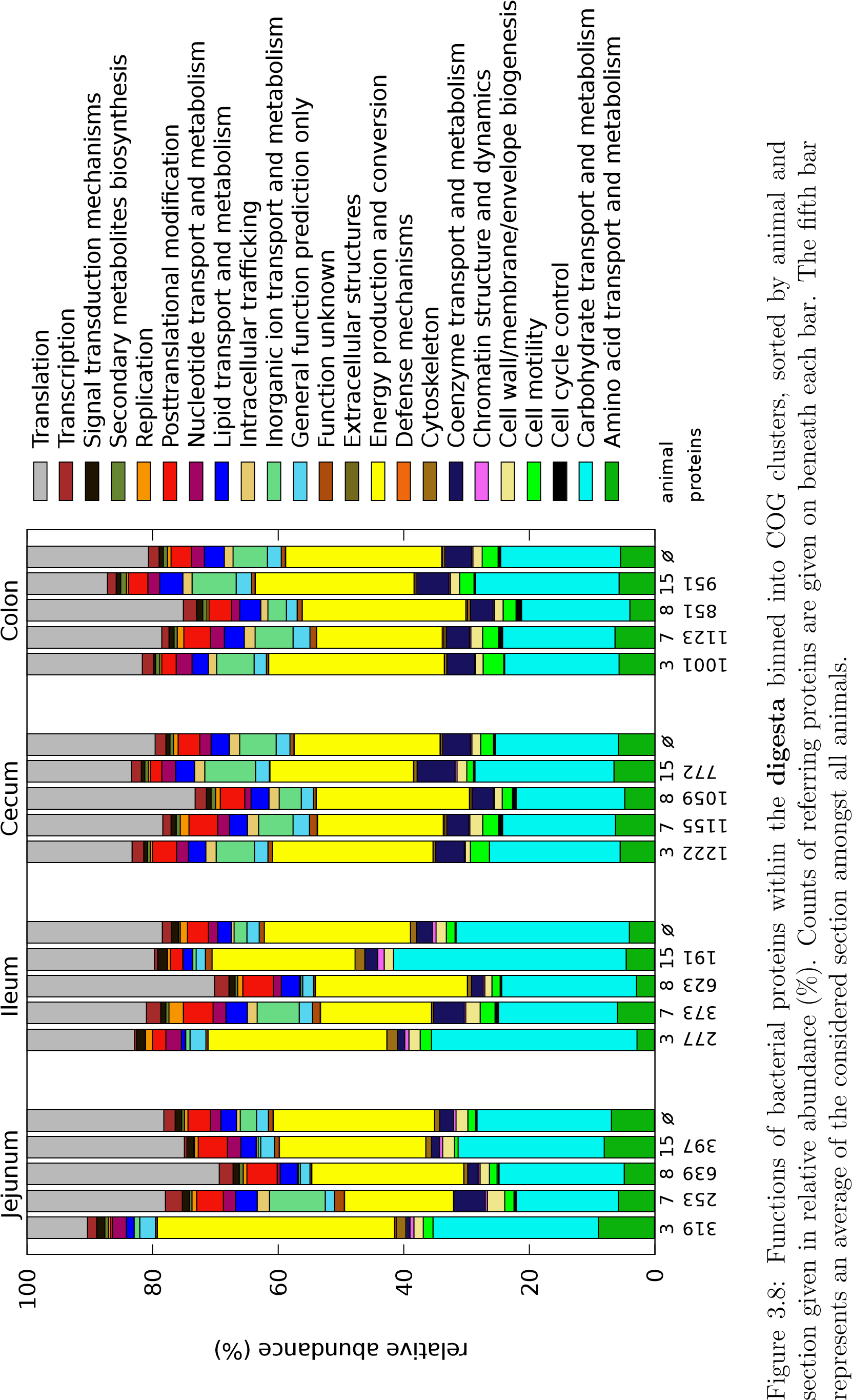


Figure S5: COG clusters of bacterial proteins identified in the porcine digesta samples sorted by animal and section and given in relative abundance (%). Counts of referring proteins are given beneath each bar. The ﬁfth bar represents an average of the considered section amongst all animals


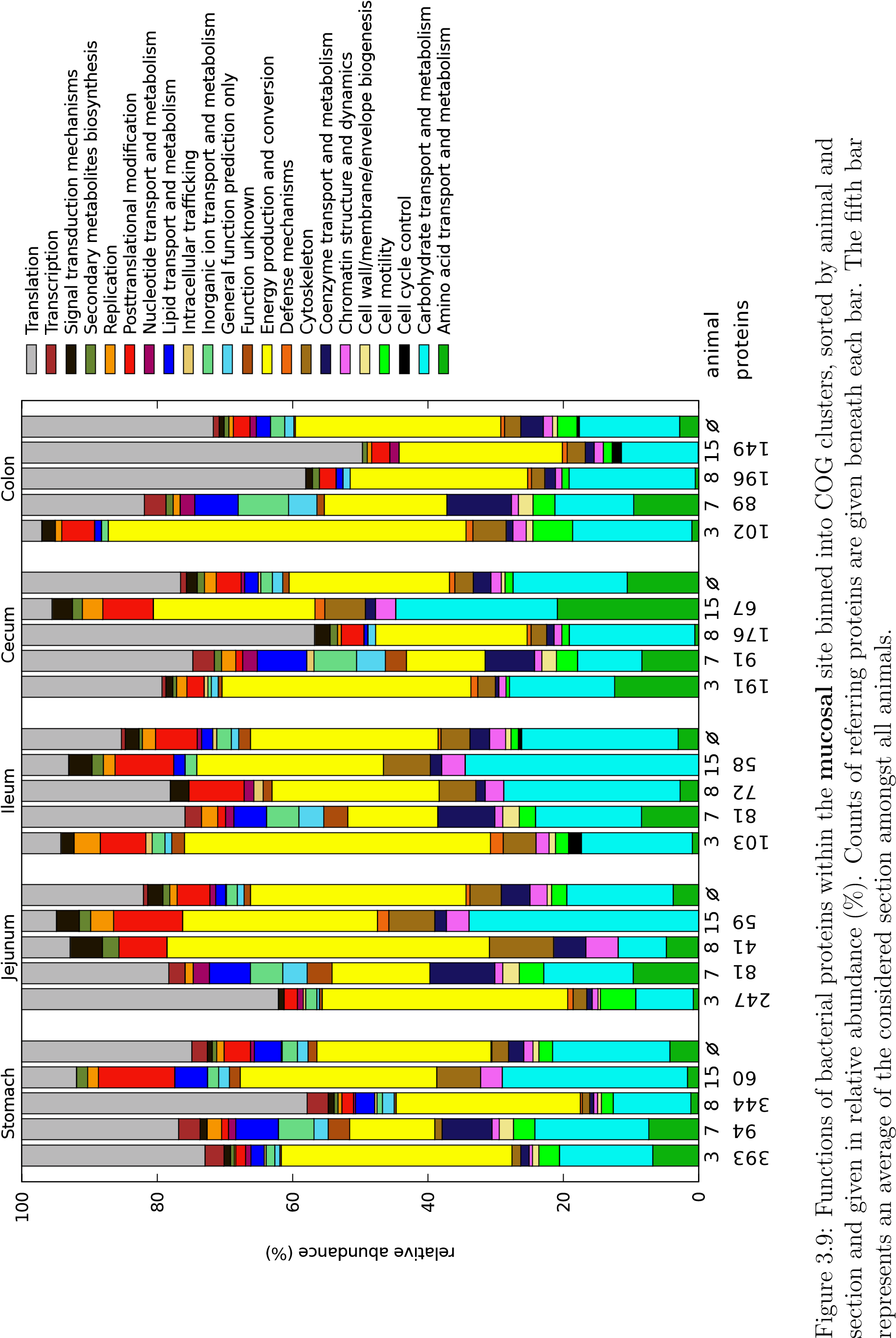


Figure S6: COG clusters of bacterial proteins identified in the porcine mucosa samples sorted by animal and section and given in relative abundance (%). Counts of referring proteins are given beneath each bar. The ﬁfth bar represents an average of the considered section amongst all animals
